# Experimental evidence rules out mosquitoes as vectors of Lyme disease

**DOI:** 10.1186/s13071-025-06823-x

**Published:** 2025-06-04

**Authors:** Miriama Pekľanská, Kateřina Kuníková, Romana Vlčková, Hana Slabová, David Hartmann, Karolina Volfová, Ivo Rudolf, Silvie Šikutová, Ryan O. M. Rego, Fernando Gabriel Noriega, Ondřej Hajdušek, Jan Perner, Jan Votýpka, Marcela Nouzová, Radek Šíma

**Affiliations:** 1 Institute of Parasitology, Biology Centre of the Czech Academy of Sciences, Branišovská 31, 370 05 České Budějovice, Czech Republic; 2 Faculty of Science, University of South Bohemia, Branišovská 1645/31a, 370 05 České Budějovice, Czech Republic; 3 Department of Parasitology, Faculty of Science, Charles University, Viničná 7, 128 44, Prague, Czech Republic; 4 Institute of Vertebrate Biology, Czech Academy of Sciences, Květná 8, 603 65 Brno, Czech Republic; 5 Department of Biological Sciences and Biomolecular Science Institute, Florida International University, Miami, FL 33199 USA; 6 Bioptic Laboratory, Mikulášské náměstí 4, 326 00 Plzeň, Czech Republic; 7 Department of Pathology, Faculty of Medicine in Plzen, Charles University, and University Hospital, E. Beneše 13, 305 99 Plzeň, Czech Republic

**Keywords:** Lyme disease, Borreliosis, Mosquito, Tick, *Borrelia*, Transmission

## Abstract

**Background:**

Lyme disease, caused by *Borrelia burgdorferi* sensu lato (s.l.), is the most common vector-borne disease in the Northern Hemisphere, with *Ixodes* ticks as its primary vectors. However, many patients do not recall tick bites, fueling speculation about alternative transmission routes, particularly via mosquito bites. This belief is reinforced by studies reporting *Borrelia* presence in mosquitoes. This study evaluates whether three mosquito species can acquire, maintain, and transmit *Borrelia* spirochetes.

**Methods:**

Mosquitoes (*Aedes aegypti*, *Culex quinquefasciatus*, and *Culex pipiens* biotype *molestus*) were fed on *Borrelia*-infected mice to assess pathogen acquisition. Additional experiments involved ex vivo feeding on *Borrelia*-enriched blood to examine spirochete persistence in the mosquito gut. The potential for mechanical transmission was tested by simulating interrupted feeding between infected and naive hosts. The role of trypsin in *Borrelia* survival and infectivity was also investigated.

**Results:**

Mosquitoes exhibited low efficiency in acquiring *Borrelia* from infected hosts. Spirochetes artificially introduced through ex vivo blood meals were rapidly eliminated during digestion, primarily due to trypsin activity. Inhibition of trypsin prolonged spirochete persistence and infectivity in the mosquito gut. Mechanical transmission experiments revealed no evidence of *Borrelia* transmission from infected to naive hosts.

**Conclusions:**

Our findings demonstrate that mosquitoes lack the biological capacity to efficiently acquire and maintain *B. burgdorferi* s.l. spirochetes and are unable to transmit them through natural or mechanical means. This study provides compelling evidence against mosquito-borne transmission of Lyme disease and reinforces *Ixodes* ticks as the sole competent vectors, which is crucial for targeted public health interventions and accurate risk communication.

**Graphical Abstract:**

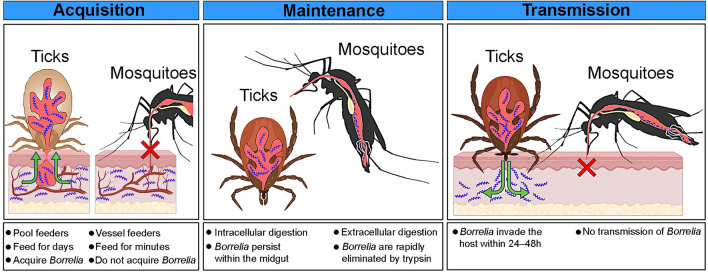

## Background

Lyme disease (borreliosis) is the most prevalent vector-borne disease in Europe and the United States, representing a significant public health concern in the Northern Hemisphere. The disease is primarily caused by spirochetes from the *Borrelia burgdorferi* sensu lato (s.l.) complex. In the United States, *B. burgdorferi* sensu stricto (s.s.) Johnson et al., 1984 is the predominant species, whereas in Europe, the most common species associated with human infections are *Borrelia afzelii* Canica et al., 1993, *Borrelia garinii* Baranton et al., 1992, and *B. burgdorferi* s.s. *Borrelia* spirochetes are maintained in nature through an enzootic cycle involving small vertebrates, primarily rodents and birds, with humans serving as incidental hosts [1].

Ticks of the genus *Ixodes* are widely recognized as the primary vectors of Lyme disease. However, many patients diagnosed with the disease do not recall being bitten by a tick [2, 3]. In a retrospective cohort study, only 56 of 210 (26.7%) Canadian Lyme disease patients reported a tick bite [4]. In another study, 1070 forestry workers from Ukraine were tested using enzyme-linked immunosorbent assay (ELISA) for specific *B. burgdorferi* immunoglobulin M (IgM) and IgG antibodies and were surveyed regarding their history of tick bites. Among those who did not recall any tick bites, 27.0% tested seropositive [5]. Similarly, data from the MyLymeData online patient registry revealed that 59% of 3903 US patients clinically diagnosed with Lyme disease either did not recall or were unsure about a tick bite [6]. This is primarily due to the painless nature of tick bites and the small size of nymphal ticks, which are responsible for most human cases of Lyme disease and often go unnoticed. In contrast, people are more likely to recall the painful bites of other blood-feeding insects, such as mosquitoes, deer flies, horse flies, and black flies. As a result, people frequently associate their Lyme disease infection with previous insect bites, perpetuating the belief that these insects can transmit the pathogen. This belief is further reinforced by numerous studies reporting the detection of *Borrelia* spirochetes in arthropods other than ticks, including mosquitoes [7–11], raising questions about their vector competence. However, the mere detection of *Borrelia* in mosquitoes does not necessarily indicate their ability to transmit the pathogen. To date, experimental evidence confirming or refuting the role of mosquitoes in transmitting Lyme disease spirochetes remains lacking.

This study investigates whether mosquitoes are capable of transmitting Lyme disease by evaluating their ability to acquire, maintain, and transmit *B. burgdorferi*, *B. afzelii*, and *B. garinii* spirochetes. Our findings provide critical insights into the vector competence of mosquitoes, addressing public health concerns and contributing to a deeper understanding of Lyme disease ecology.

## Methods

### *Borrelia* spirochetes and laboratory animals

Infectious and low-passage strains of *B. afzelii* CB43 [12, 13], *B. burgdorferi* s.s. N40 (isolate obtained from Prof. Joppe W.R. Hovius, Amsterdam University Medical Center, Netherlands), and *B. garinii* WSLB 8096/1 (isolate obtained from Dr. Jiří Nepeřený, Bioveta, Czech Republic) were grown in BSK-H medium (Sigma-Aldrich, St. Louis, MO, USA) at 33 °C. A low-passage strain of the relapsing fever spirochete *Borrelia duttonii* Novy & Knapp, 1906, strain 1120 K3 (origin, Congo) [14], was grown in mBSK medium supplemented with 10% rabbit serum at 35 °C [15]. For both mouse injections and acquisition experiments using artificial membrane feeders, spirochetes were counted using dark-field microscopy.

The mosquito colonies were obtained from the Laboratory of Molecular Biology and Physiology of Mosquitoes, Institute of Parasitology, Biology Centre, Czech Academy of Sciences (*Aedes aegypti* Linnaeus, 1762) and from the Faculty of Science, Charles University in Prague (*Culex quinquefasciatus* Say, 1823 and *Culex pipiens* biotype *molestus* Forskal, 1775). The mosquitoes were kept in 20 × 20 × 20 cm nylon nets in an incubator with constant conditions of 28 °C, 80% humidity and 16 h:8 h light/dark photoperiod. The mosquitoes had permanent access to 10% sucrose. The mosquito larvae were fed aquarium fish food (*Culex* spp: TetraMin flakes; *Ae. aegypti*: TabiMin tablets, Tetra, Melle, Germany). Female mosquitoes were used for experiments 1 week after eclosion. *Ixodes ricinus* Linnaeus, 1758 ticks were obtained from the Institute of Parasitology, Biology Centre, Czech Academy of Sciences. They were maintained under controlled conditions (temperature 24 °C, 95% humidity, 15 h:9 h light/dark photoperiod). *Borrelia afzelii*- and *B. burgdorferi* s.s.-infected nymphs were prepared as described previously [12]. C3H/HeN mice (Jackson Laboratory, Bar Harbor, ME, USA) were used for mosquito feeding, as well as *Borrelia* acquisition and transmission experiments.

All laboratory animals were treated in accordance with the Animal Protection Law of the Czech Republic no. 246/1992 Sb., ethics approval no. 25/2020. The study was approved by the Institute of Parasitology, Biology Centre CAS and the Central Committee for Animal Welfare, Czech Republic (Protocol No. 25/2020).

### PCR detection and quantification of *Borrelia*

DNA was extracted using the NucleoSpin Tissue kit (Macherey-Nagel, Düren, Germany) following the manufacturer’s protocol. Spirochete detection in mosquitoes and murine tissues was performed by a nested polymerase chain reaction (PCR) targeting a 222-base-pair (bp) fragment of the 23S rRNA gene. Quantitative real-time PCR (qPCR) was employed to quantify the total spirochete load in mosquitoes by amplifying a 154-bp fragment of the flagellin gene. The PCR and qPCR amplification conditions followed previously described protocols [12].

### Mouse acquisition experiments

Six-week-old female C3H/HeN mice were infected by subcutaneous injection of 10^5^ *B. afzelii* or *B. burgdorferi* s.s. spirochetes per mouse. The presence of spirochetes in ear biopsies was verified by PCR 3 weeks post-injection. The *Borrelia*-positive mouse was then anesthetized and placed into the mosquito net, allowing *Ae. aegypti*, *Cx. quinquefasciatus, *and* Cx. pipiens* biotype *molestus* mosquitoes to feed ad libitum on the mouse.

Eight-week-old female C3H/HeN mice were inoculated intraperitoneally and subcutaneously with 10^5^ *B. duttonii* spirochetes per mouse. On days 3 and 4 post-inoculation, 5 μl of blood from tail snips was placed on glass slides to confirm the presence of spirochetes by dark-field microscopy. Once spirochetemia was assured, the mice were used to feed *Ae. aegypti*.

Twenty fully engorged mosquitoes were collected from each experimental group and tested for the presence of spirochetes by PCR.

### Acquisition experiments on artificial membrane feeders

The 3D-printed feeders were designed de novo in FreeCAD 0.20 based on previously published nano-feeders [16] and printed on a Creality Halot Sky (CL-89) 3D printer with low-odor rigid resin (Creality, Shenzhen, China), with the following modifications: (i) the blood reservoir capacity was increased to 1 ml, (ii) the feeding area was expanded to 2.4 cm^2^, (iii) the blood filling port was shaped to tightly accommodate a 1 ml pipette tip, and (iv) the warm water circulation connectors were made compatible with 5 mm internal diameter silicone tubing.

A total of 3.5 × 10^7^ *B. afzelii*, *B. burgdorferi* s.s., and *B. garinii* spirochetes were resuspended in 1 ml blood (mouse blood for *B. afzelii* and *B. burgdorferi* s.s.; and chicken blood for *B. garinii*) supplemented with ATP (1 mM) and injected into the feeding unit. The blood temperature was maintained at 37 °C. Prior to feeding, *Ae. aegypti* and *Cx. quinquefasciatus* mosquitoes were deprived of sugar for 12 h and then transferred to feeding units using an aspirator. Mosquitoes were allowed to feed until fully satiated. At 0, 24, 48, 72, and 96 h post-feeding, mosquitoes were euthanized by snap-freezing, and the presence of spirochetes in whole-body homogenates was assessed using nested PCR and quantified by qPCR. For each mosquito species and time point combination, 20 mosquitoes were tested.

### *Borrelia* viability and infectivity experiments

Female *Ae. aegypti* and *Cx. quinquefasciatus* were fed on membrane feeding units containing mouse blood spiked with *B. afzelii* spirochetes. Whole-body homogenates were prepared from mosquitoes at 0, 24, 48, and 72 h post-feeding and subcutaneously injected into C3H/HeN mice (five mice/group, one mosquito/mouse). Four weeks after injection, infection in ear, heart, and bladder tissues was determined by nested PCR.

### Effect of trypsin on *Borrelia* survival

Female mosquitoes were fed on membrane feeding units containing mouse blood spiked with *B. afzelii* spirochetes and soybean trypsin inhibitor (SBTI) at a concentration of 2 mg/ml for *Cx. quinquefasciatus* and 1 mg/ml for *Ae. aegypti*. Mosquitoes were allowed to feed until fully satiated. At 0, 24, 48, 72, and 96 h post-feeding, mosquitoes were euthanized by snap-freezing, and the presence of spirochetes in whole-body homogenates was assessed using nested PCR and quantified by qPCR. For each mosquito species and time point combination, 20 mosquitoes were tested. The results were compared to untreated control mosquitoes.

To assess the direct impact of trypsin on *Borrelia* viability, cultures of *B. afzelii* (3.5 × 10^7^ spirochetes/ml) were treated with trypsin (300 ng/ml). Control cultures were treated with heat-inactivated trypsin (10 min at 95 °C). The viability of *B. afzelii* spirochetes was evaluated after 48 h.

### Transmission experiments

Female *Ae. aegypti* and *Cx. quinquefasciatus* mosquitoes were fed on membrane feeding units containing mouse blood spiked with *B. afzelii* or *B. burgdorferi* s.s. spirochetes. After digestion and egg-laying, the females were fed on naive mice (four mice/group, 15 mosquitoes/mouse). Immediately after feeding, DNA was extracted from fully engorged mosquitoes. Four weeks after the second feeding, ear, skin, bladder, and heart biopsies were collected and the presence of *Borrelia* spirochetes in mouse tissue and mosquito samples was determined by nested PCR.

### Mechanical transmission experiments

Female *Ae. aegypti* and *Cx. quinquefasciatus* mosquitoes were fed on membrane feeding units containing mouse blood spiked with *B. afzelii* or *B. burgdorferi* s.s. spirochetes, interrupted mid-feeding, and then immediately placed on naive mice to resume their feeding (four mice/group, 18–20 mosquitoes/mouse). After feeding, DNA was extracted from the collected mosquitoes. Four weeks post-feeding, mouse tissues were collected as described above. The presence of *Borrelia* spirochetes in mouse tissue and mosquito samples was determined by nested PCR.

### Statistics and software

Data were analyzed using GraphPad Prism 10, and an unpaired Student *t*-test was used to assess statistical significance. A *P*-value of less than 0.05 was considered statistically significant. The error bars in the graphs represent the standard errors of the means.

## Results

### Mosquitoes rarely acquire *Borrelia* from infected host

To investigate whether mosquitoes can acquire *Borrelia* spirochetes from infected hosts, female *Ae. aegypti*, *Cx. quinquefasciatus*, and *Cx. pipiens* biotype *molestus* mosquitoes were fed on mice infected with *B. afzelii* or *B. burgdorferi* s.s. PCR analysis showed that 15% of *Ae. aegypti* (3/20) fed on *B. burgdorferi* s.s.-infected mice tested positive, whereas no spirochetes were detected in *Ae. aegypti* that fed on mice infected with *B. afzelii*, or in *Cx. quinquefasciatus* or *Cx. pipiens* biotype *molestus* fed on mice infected with either *Borrelia* species (Table 1).

**Table 1:** Acquisition of *Borrelia* spirochetes by mosquitoes

	*B. afzelii*	*B. burgdorferi*	*B. duttonii*
*Ae. aegypti*	0% (0/20)	15% (3/20)	100% (20/20)
*Cx. quinquefasciatus*	0% (0/20)	0% (0/20)	ND
*Cx. pipiens molestus*	0% (0/20)	0% (0/20)	ND

*Ae. aegypti*, *Cx. quinquefasciatus*, and *Cx. pipiens* biotype *molestus* females were fed on mice infected with *B. afzelii* or *B. burgdorferi* s.s. Twenty fully engorged mosquitoes were collected from each experimental group and tested for the presence of spirochetes using PCR. As a control for spirochete acquisition, *Ae. aegypti* females were fed on mice infected with *B. duttonii*. *ND* not done

In a control experiment using *B. duttonii*, a *Borrelia* species naturally present in the bloodstream, all *Ae. aegypti* (20/20) tested positive for spirochetes 24 h post-feeding (Table 1).

These findings indicate that, unlike ticks, mosquitoes lack mechanisms to actively attract *Borrelia* to their feeding sites. Instead, the spirochetes must already be present in the bloodstream for potential mosquito ingestion. Overall, these results indicate a low probability of mosquitoes acquiring the Lyme disease pathogen under natural conditions.

### *Borrelia* spirochetes are quickly eliminated during mosquito digestion

Previous experiments demonstrated that mosquitoes can only rarely acquire *Borrelia* spirochetes. We further investigated the fate of *Borrelia* spirochetes in mosquitoes. *Aedes aegypti* and *Cx. quinquefasciatus* were fed on membrane feeding units containing mouse blood spiked with *B. afzelii*, *B. burgdorferi* s.s., or *B. garinii*. Spirochetes were detected and quantified by PCR and qPCR at 0, 24, 48, 72, and 96 h post-feeding.

**Fig. 1:**
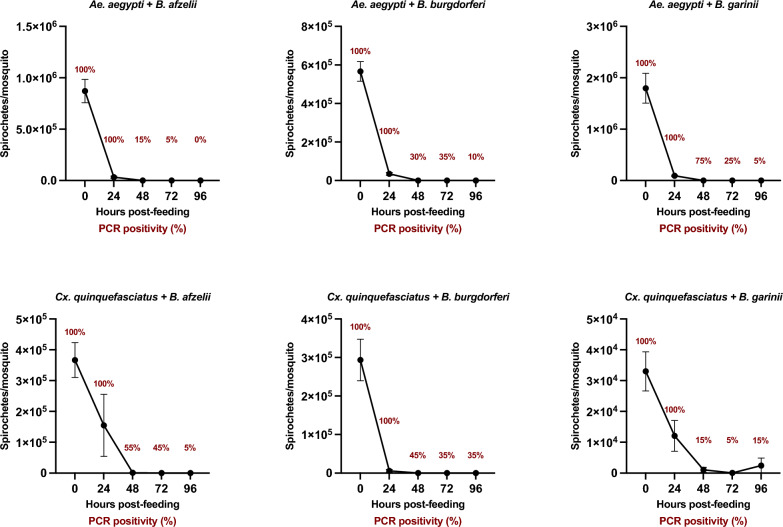
Decline in *Borrelia* spirochete numbers in mosquitoes during post-blood meal digestion. The temporal dynamics of *Borrelia* spirochete levels in *Ae. aegypti* and *Cx. quinquefasciatus* following ingestion of mouse blood spiked with *B. afzelii*, *B. burgdorferi* s.s., or *B. garinii*. Spirochetes were detected using PCR and quantified by qPCR at 0, 24, 48, 72, and 96 h post-feeding. Error bars represent the standard error of the mean

Spirochetes were successfully introduced into mosquitoes via artificial feeding, with 100% efficacy. By 24 h, spirochete DNA remained detectable in all mosquitoes but gradually disappeared over subsequent time points. In *Cx. quinquefasciatus*, spirochete decline was slower than in *Ae. aegypti*. At 96 h, 5%, 35%, and 15% of *Cx. quinquefasciatus* and 0%, 10%, and 5% of *Ae. aegypti* were PCR-positive for *B. afzelii*, *B. burgdorferi* s.s., and *B. garinii*, respectively (Fig. 1).

A rapid decline in spirochete numbers post-feeding was confirmed by qPCR. Within 24 h, median spirochete counts in *Ae. aegypti* decreased 58-, 17-, and 23-fold for *B. afzelii*, *B. burgdorferi* s.s., and *B. garinii*, respectively, with near-total elimination by 96 h (two mosquitoes remained positive for *B. burgdorferi* s.s. and one for *B. garinii*). In *Cx. quinquefasciatus*, declines at 24 h were 8-, 213-, and fourfold, and 5/30 mosquitoes remained positive at 96 h (three for *B. burgdorferi* s.s. and two for *B. garinii*) (Fig. 1).

These results confirm that ingested *Borrelia* spirochetes are rapidly eliminated in the mosquito gut.

### Trypsin promotes *Borrelia* clearance in mosquitoes

The previous experiment showed that *Borrelia* spirochetes are efficiently eliminated from the mosquito gut after a blood meal. Midgut trypsin plays a key role in the digestion of blood components between blood meals.

**Fig. 2:**
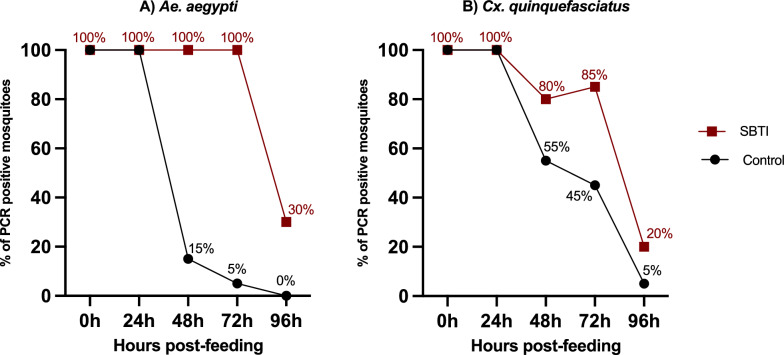
Persistence of *B. afzelii* in trypsin-inhibited mosquitoes. **A** *Aedes aegypti* and **B** *Cx. quinquefasciatus* were fed on mouse blood spiked with *B. afzelii* in the presence (SBTI) or absence (control) of soybean trypsin inhibitor. The presence of spirochetes in mosquitoes was assessed using PCR at 24, 48, 72, and 96 h post-feeding.

To gain deeper insight into the role of extracellular digestion in *Borrelia* survival, we next examined whether trypsin activity influences the viability of *Borrelia* spirochetes within the mosquito gut. *Aedes aegypti* and *Cx. quinquefasciatus* were fed *B. afzelii*-spiked blood with or without soybean trypsin inhibitor (SBTI). Trypsin inactivation prolonged *B. afzelii* persistence in both mosquito species. At 72 h post-feeding, spirochetes were detected in 100% (20/20) of trypsin-inactivated *Ae. aegypti*, compared to 5% (1/20) of control mosquitoes (Fig. 2A). Similarly, *B. afzelii* persisted longer in *Cx. quinquefasciatus* mosquitoes with inactivated trypsin. At 72 h post-feeding, 85% (17/20) of SBTI-treated *Cx. quinquefasciatus* mosquitoes were positive, compared to 45% (9/20) of control mosquitoes (Fig. 2B).

In vitro assays confirmed the direct effect of trypsin on *B. afzelii*. Cultures treated with active trypsin showed ~ 97% spirochete death at 48 h, compared to ~ 34% with heat-inactivated trypsin. These findings establish trypsin as a critical factor in *Borrelia* clearance.

### *Borrelia* lose infectivity during mosquito digestion

The infectivity of *B. afzelii* during digestion was assessed using homogenates of fed mosquitoes. All mice (5/5) injected with *Ae. aegypti* homogenates at 0 h post-feeding became infected. At 24 h post-feeding, spirochetes remained infectious for 4/5 mice, while mice injected with homogenates at 48 and 72 h post-feeding tested negative. Similarly, 5/5, 4/5, 2/5, and 0/5 mice injected with *Cx. quinquefasciatus* homogenates at 0, 24, 48, and 72 h post-feeding, respectively, developed infections.

Trypsin inhibition extended spirochete infectivity. At 72 h, 3/5 mice injected with homogenates from trypsin-inhibited *Cx. quinquefasciatus* developed infections, compared to 0/5 in controls (Table 2).

**Table 2:** Infectivity of *B. afzelii* during mosquito digestion

	*Ae. aegypti*				*Cx. quinquefasciatus*			
	0 h	24 h	48 h	72 h	0 h	24 h	48 h	72 h
Control	5/5	4/5	0/5	0/5	5/5	4/5	2/5	0/5
SBTI	5/5	ND	1/5	0/5	5/5	ND	3/5	3/5

These results indicate that *Borrelia* spirochetes survive in the mosquito gut for up to 48 h but lose their infectivity thereafter.

*Aedes aegypti* and *Cx. quinquefasciatus* were fed on mouse blood spiked with *B. afzelii* in the presence (SBTI) or absence (control) of soybean trypsin inhibitor. Homogenates of mosquitoes collected at 0, 24, 48, and 72 h post-feeding were injected subcutaneously into mice (five mice/group). Infection in murine tissues was assessed using nested PCR 4 weeks after injection.

### Mosquitoes cannot naturally transmit *Borrelia*

We further tested whether *Borrelia* spirochetes, if surviving mosquito digestion, could be transmitted during subsequent feeding. To model this, *Ae. aegypti* and *Cx. quinquefasciatus* were fed *B. afzelii*- or *B. burgdorferi* s.s.-spiked blood, allowed to digest and oviposit, and then fed on naive mice.

No spirochetes were detected in any mice (0/4) exposed to mosquitoes, or in the fully fed mosquitoes collected post-feeding (Table 3A), confirming that *Borrelia* cannot survive in mosquitoes between blood meals. In contrast, all (4/4) control mice exposed to infected *I. ricinus* ticks became infected, validating the efficiency of tick transmission under identical experimental conditions.

**Table 3:** Transmission of *Borrelia* spirochetes by feeding of infected mosquitoes or *I. ricinus* nymphs

	(A) Natural transmission		(B) Mechanical transmission	
	*B. afzelii*	*B. burgdorferi*	*B. afzelii*	*B. burgdorferi*
*Ae. aegypti*	0/4	0/4	0/4	0/4
*Cx. quinquefasciatus*	0/4	0/4	0/4	0/4
*I. ricinus*	4/4	4/4	ND	ND

(A) *Borrelia* transmission via natural feeding. (B) *Borrelia* transmission via interrupted feeding. Numbers represent the number of infected mice/total number of experimental mice in each group. *ND* not done

### *Borrelia* cannot be transmitted mechanically by mosquitoes

Next, we tested whether interrupted feeding could result in mechanical transmission of *Borrelia* spirochetes. This hypothesis suggests that mosquitoes interrupted while feeding on an infected host could transmit spirochetes adhered to their mouthparts to a new host during subsequent feeding. *Aedes aegypti* and *Cx. quinquefasciatus* were fed on *B. afzelii*- or *B. burgdorferi* s.s.-spiked blood, interrupted mid-meal, and immediately placed on naive mice to resume feeding.

No *B. afzelii* or *B. burgdorferi* s.s. infections were detected in the exposed mice (0/4), although all mosquitoes collected post-feeding tested positive for spirochetes (Table 3B).

These findings confirm that mosquitoes cannot transmit *Borrelia* spirochetes via mechanical or natural routes.

## Discussion

In recent years, there has been growing interest and concern regarding the potential role of mosquitoes in the transmission of Lyme disease. While ticks have long been recognized as the primary vectors for *B. burgdorferi* s.l., the causative agent of Lyme disease, emerging evidence suggests that other arthropods, including mosquitoes, may also play a role in the transmission cycle. The presence of spirochetes in mosquitoes was first reported in 1907 by Jaffé, who microscopically observed a spirochete in *Culex* sp. [17]. Magnarelli and Anderson later detected *B. burgdorferi* s.s. by indirect fluorescent-antibody staining in *Aedes* mosquitoes (with a prevalence of up to 11.1%), three species of horse flies (up to 14.3%), and four species of deer flies (up to 10.5%) [18]. More recently, Melaun et al. detected DNA from *B. afzelii*, *Borrelia bavariensis* Margos et al., 2013, and *B. garinii* in 10 mosquito species from four different genera: *Aedes* (incl. *Ochlerotatus*), *Culiseta*, and *Culex* [11]. However, the mere detection of *Borrelia* in mosquitoes does not necessarily imply their ability to effectively transmit the pathogen to humans or other vertebrate hosts. In this study, we experimentally tested the ability of three mosquito species to transmit Lyme disease spirochetes.

Vector competence is the inherent capability of an organism to acquire, maintain, and transmit a specific pathogen. Ticks of the genus *Ixodes*, the competent vectors of Lyme disease, fulfil all these requirements. They remain attached to a host for an extended period, typically spanning several days, creating favorable conditions for the acquisition of *B. burgdorferi* s.l. During this time, spirochetes migrate from the host tissues into the feeding cavity that forms in the skin and are then ingested by the tick [12, 19]. In contrast, mosquitoes feed from blood capillaries and do so much faster than ticks, typically within seconds to minutes [20]. This rapid feeding behavior limits their ability to effectively acquire spirochetes, as demonstrated in our study, where only 15% of *Ae. aegypti* mosquitoes feeding on *B. burgdorferi* s.s.-infected mice tested positive for spirochetes. In addition, no spirochetes were detected in *Ae. aegypti* exposed to *B. afzelii*-infected mice, or in *Cx. quinquefasciatus* or *Cx. pipiens* biotype *molestus* mosquitoes feeding on mice infected with either *B. afzelii* or *B. burgdorferi* s.s.

In contrast, 100% of mosquitoes feeding on *B. duttonii*-infected mice ingested the spirochetes. *Borrelia duttonii*, the causative agent of tick-borne relapsing fever, is transmitted by the argasid tick *Ornithodoros moubata* Murray, 1877. Unlike ixodid ticks, argasid ticks feed rapidly (in under 10 min), making their feeding behavior more similar to that of mosquitoes. *Borrelia duttonii* spirochetes are well adapted to this rapid feeding. Compared to *B. burgdorferi* s.l., which are rarely found in the blood [21], *B. duttonii* are present in high concentrations in the blood during the febrile phase of the disease (up to 10^7^ spirochetes/ml) [22].

Overall, these findings indicate that mosquitoes have a low probability of being infected with *B. burgdorferi* s.l., likely due to the adaptation of these spirochetes to the slow feeding behavior of ixodid ticks and their absence in the blood.

The next biological factor influencing vector competence is the ability to maintain pathogens between blood meals. *Ixodes* ticks are highly competent in maintaining *B. burgdorferi* s.l. spirochetes. *Ixodes* larvae initially acquire a small number of *Borrelia* spirochetes, which colonize and multiply in the tick midgut post-repletion [12, 19]. After molting into nymphs, the spirochetes adhere to the midgut epithelial cells [23] and remain there until the next feeding. *Borrelia burgdorferi* s.l. spirochetes have evolved mechanisms to survive in the nutrient-poor tick gut during long intervals between blood meals.

Little is known about the competence of mosquitoes to maintain *B. burgdorferi* s.l. spirochetes. Magnarelli et al. demonstrated that *B. burgdorferi* s.s. can survive briefly in *Aedes* mosquitoes after feeding on spirochete-spiked bovine blood. However, the spirochetes were quickly eliminated and rarely detectable in *Ae. aegypti* and *Ae. triseriatus* Say, 1823 up to 14 days post-feeding [24].

In our study, artificial membrane feeding efficiently introduced *Borrelia* spirochetes into the mosquito gut. However, the spirochetes were rapidly cleared during the post-feeding period. This clearance was driven by the digestion mechanisms in the mosquito midgut. Although both mosquitoes and ticks feed on blood, they have developed different digestive mechanisms. Tick digestion is intracellular. Once blood is ingested, it is absorbed into midgut cells where lysosomes degrade blood components using multiple proteolytic enzymes [25]. In mosquitoes, however, digestion is extracellular. Blood enters the mosquito midgut, which is the primary site of digestion. The blood is degraded by digestive enzymes secreted by the midgut epithelium. Trypsin plays a central role in this process [26]. In our experiments, inactivation of trypsin significantly prolonged the persistence of spirochetes in the midgut of mosquitoes, highlighting the importance of extracellular digestion in preventing *Borrelia* maintenance.

The final requirement of the vector competence is the ability to transmit the pathogen to a new host during subsequent feeding. Studies on ticks have shown that the infectivity of *Borrelia* spirochetes depends on differential gene expression stimulated by feeding [12, 27]. Furthermore, to establish an infection in the host, *Borrelia* spirochetes must evade the tick midgut and migrate to the feeding lesion. These processes typically require 24–48 h [12].

Unlike ticks, mosquitoes only need a few minutes to complete a blood meal [20]. This short feeding period limits the time available for *Borrelia* spirochetes to transition from a non-infectious to a vertebrate-infective state. In addition, *Borrelia* would need to exit the mosquito midgut, traverse the hemocoel, and reach the salivary glands. This process would require additional time and multiple adaptations, including evasion of immune defenses in the mosquito hemolymph, receptor-mediated infection of the salivary glands, and other mechanisms that typically evolve over long periods of coevolution [28]. Supporting this hypothesis, a transmission experiment demonstrated that *Ae. aegypti* and *Cx. quinquefasciatus* mosquitoes experimentally infected with *B. afzelii* and *B. burgdorferi* s.s. spirochetes were unable to transmit the infection to naive mice.

Mechanical transmission has also been considered as a potential route for mosquito-mediated *Borrelia* transmission. In this scenario, a mosquito would feed on an infected host, be disturbed, and then complete feeding on another host, mechanically transferring the spirochetes adhering to its mouthparts to the second host. However, an experiment in which mosquitoes were half-fed on mouse blood spiked with *B. afzelii* or *B. burgdorferi* s.s. spirochetes, and then allowed to complete feeding on naive mice, ruled out this possibility. Despite the presence of spirochetes in the mosquitoes, there was no transmission to the second host.

## Conclusions

Our study reinforces the consensus that mosquitoes cannot serve as competent vectors for Lyme disease spirochetes, underscoring the exclusive role of ixodid ticks in transmitting this pathogen. Specifically, (1) mosquitoes are unable to acquire sufficient quantities of spirochetes during feeding; (2) ingested spirochetes are efficiently eliminated during blood meal digestion, primarily due to the activity of trypsin; and (3) mosquitoes cannot serve as either natural or mechanical vectors for *Borrelia* spirochetes. Our findings highlight the need for careful interpretation when evaluating the presence of *Borrelia* in non-tick arthropods. Attributing vectorial capacity to mosquitoes based solely on pathogen detection risks failing to capture the complex biological and ecological requirements for Lyme disease transmission.

## Data Availability

No datasets were generated or analyzed during the current study.
